# In vitro Analysis of Stalled Ribosomes Using Puromycin Incorporation

**DOI:** 10.21769/BioProtoc.4744

**Published:** 2023-08-20

**Authors:** MaKenzie R. Scarpitti, Michael G. Kearse

**Affiliations:** 1 The Biomedical Sciences Graduate Program, The Ohio State University, Columbus, OH, USA; 2 Department of Biological Chemistry and Pharmacology, The Ohio State University, Columbus, OH, USA; 3 Center for RNA Biology, The Ohio State University, Columbus, OH, USA

**Keywords:** Cycloheximide, Elongation inhibition, Polysomes, Protein synthesis, Sucrose cushion, Ultracentrifugation

## Abstract

Ribosome footprint profiling has demonstrated that ribosomes can be slowed or stalled on select mRNAs, often due to the presence of rare codons, stalling motifs, or via a ribosome-binding protein (e.g., FMRP). Stalled ribosomes can act as physical roadblocks for trailing ribosomes and ultimately can cause ribosome collisions that stimulate no-go mRNA decay. Detecting stalled or slowed ribosomes in cells by ribosome footprint profiling or classic polysome profiling is laborious, technically challenging, and low throughput. Here, we present a protocol to assay for stalled ribosomes on in vitro–transcribed reporter mRNAs using a robust, commercially available mammalian in vitro translation lysate and an optimized low-speed sucrose cushion. In short, we take advantage of the ability of puromycin to incorporate into the nascent polypeptide and cause the ribosome to dissociate from the mRNA during active elongation, as well as the ability to selectively pellet ribosomes through a low-speed sucrose cushion due to their large molecular weight. Stalled ribosomes are not actively elongating and do not incorporate puromycin, allowing the ribosome-bound mRNA to pellet in the low-speed sucrose cushion. RT-qPCR is used to quantify the amount of ribosome-bound reporter mRNA in the pellet. This workflow allows for direct assessment of stalled ribosomes and is fully amendable to insertion of putative stalling motifs in the target mRNA, as well as supplementation with recombinant proteins or small molecule inhibitors that target translation elongation.

Key features

This protocol is optimized for cap-dependent in vitro translation in the dynamic linear range.

Details for generating capped reporter mRNA in one day are provided.

Requires as little as one day to complete if starting with in vitro–transcribed mRNA.

This protocol requires access to an ultracentrifuge and a real-time PCR system.

**Graphical overview**


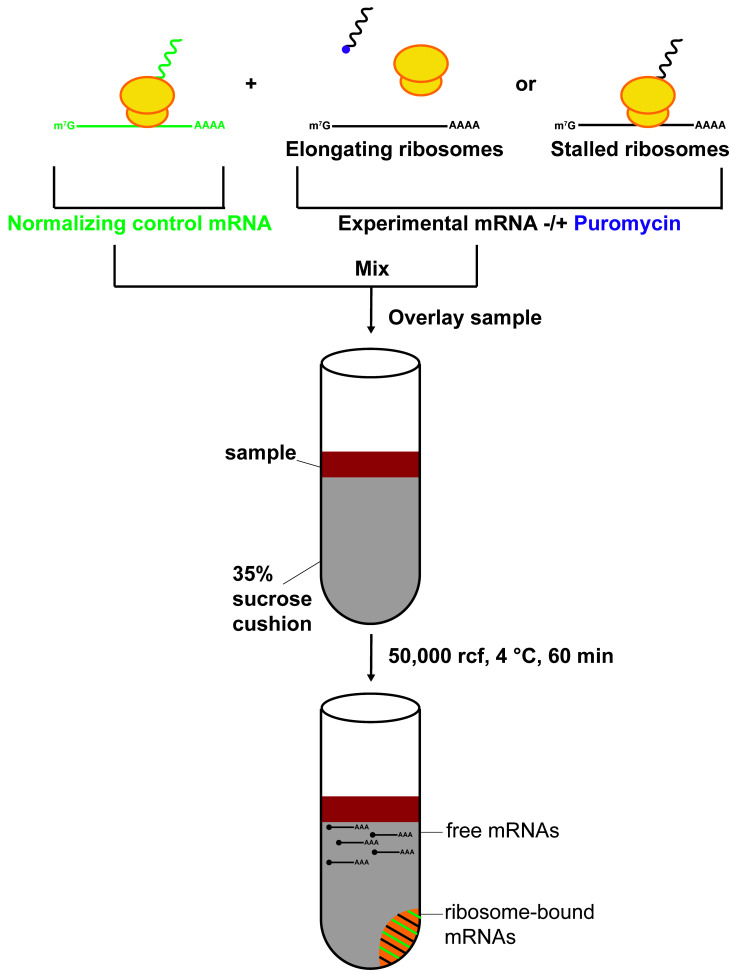


## Background

Upon initiating at a start codon, ribosomes proceed through elongation with repetitive cycles of decoding and translocation until they terminate at one of three stop codons (UAA, UAG, or UGA) (Dever et al., 2018). Elongating ribosomes may encounter a multitude of challenges, including rare codons, premature polyadenylation, truncated or damaged mRNA, proline-rich stalling motifs, strong or highly ordered mRNA structure, or mRNA- and ribosome-binding proteins (e.g., FMRP) (Kim and Zaher, 2022). These obstacles can stall ribosomes, which act as physical roadblocks for trailing ribosomes, resulting in ribosome collisions that stimulate the no-go mRNA decay pathway. In yeast, collided ribosomes are recognized by the E3 ligase Hel2 (ZNF598 in human and *Caenorhabditis elegans*) (Monem et al., 2023), which ubiquitinates ribosomal proteins eS10 and uS10 (Juszkiewicz et al., 2018). In yeast, ubiquitinated and collided ribosomes serve as a unique binding site for the endonuclease Cue2 (N4BP2 in humans, NONU1 in *Caenorhabditis elegans*) (D’Orazio et al., 2019). Subsequently, the new 5′ and 3′ ends are susceptible to degradation by Xrn1 and the exosome, respectively. Ribosome collisions also drive mRNA-specific feedback translation initiation inhibition to further prevent synthesis of deleterious truncated proteins (Juszkiewicz et al., 2020; Sinha et al., 2020). Upon large-scale ribosome collisions, global translation initiation is inhibited by activation of GCN2-mediated eIF2α phosphorylation (Wu et al., 2020).

Ribosome footprint profiling and classic polysome profiling can be used to detect stalled ribosomes; however, these approaches can be technically challenging, laborious, and rather low throughput. Additionally, ribosome profiling is not cost-effective when testing multiple specific mutations within reporter mRNAs or effector proteins. Here, we present a validated protocol that can be used to assess ribosome stalling in vitro that is medium-to-high throughput and can be performed in as little as one day if starting with in vitro–transcribed mRNA.

**Figure 1.**
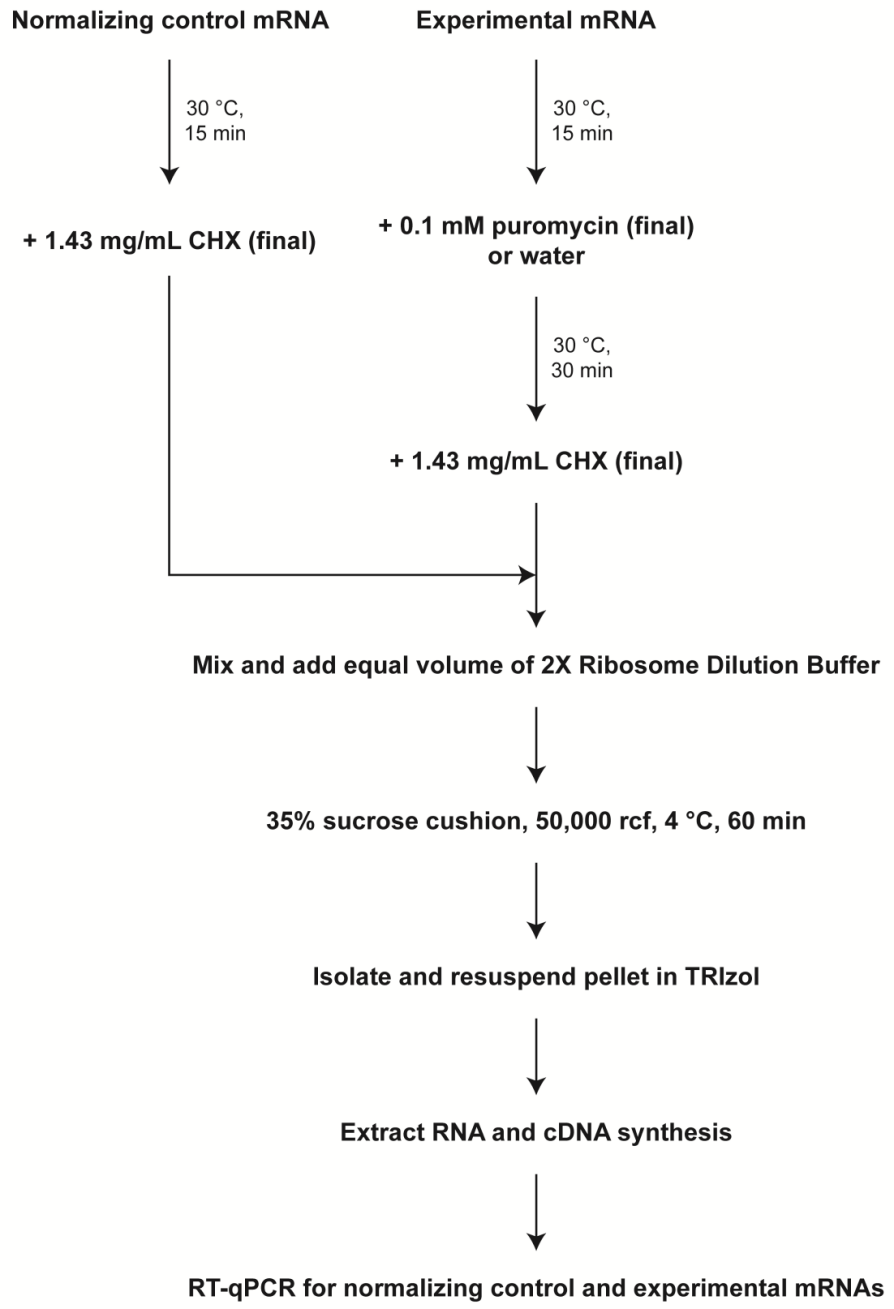
Schematic of overall strategy to assay for stalled ribosomes using puromycin incorporation and an optimized low-speed sucrose cushion. CHX = Cycloheximide.

We take advantage of the selective nature of puromycin, an amino-acyl transfer RNA analog to incorporate into nascent polypeptides of only actively elongating ribosomes (Yarmolinsky and Haba, 1959). Puromycin incorporation results in ribosomes releasing both the nascent protein and the mRNA, resulting in the collapse of polysomes to monosomes (Yarmolinsky and Haba, 1959; Azzam and Algranati, 1973; Stefani et al., 2004; Sivan et al., 2007). Using an optimized low-speed sucrose cushion and subsequent RT-qPCR, we quantify ribosome-bound reporter mRNA to determine efficacy of puromycin to dissociate ribosomes. Ribosomes that are stalled during elongation do not actively incorporate puromycin; thus, these ribosomes are insensitive to puromycin and consistently pellet bound mRNA in the presence of puromycin. This protocol utilizes a commercially available mammalian translation lysate in conditions that allow for translation in the dynamic linear range and showcases cap and scanning dependency (Kearse et al., 2016). A key feature is an independent reporter that is translated and not treated with puromycin, which serves as a normalizing control for both the low-speed sucrose cushion and RT-qPCR. The low-speed sucrose cushion has been optimized so that only ribosome-bound mRNA is pelleted. Untranslated mRNA does not have sufficient molecular weight to pellet in these conditions. We have published this strategy and validated its efficacy using the Fragile X protein FMRP (an mRNA- and ribosome-binding protein that stalls ribosomes) (Darnell et al., 2011), along with FireFly Luciferase mRNA as the normalizing control reporter and nanoLuciferase mRNA as the experimental reporter (Figure 1) (Scarpitti et al., 2022).

## Materials and reagents

**Reagents**

1. 2-Propanol (isopropanol) (Fisher Chemical, catalog number: A416P-4)
2. 3′-O-Me-m7G(5′)ppp(5′)G RNA Cap Structure Analog (anti-reverse cap analog; ARCA) (New England Biolabs, catalog number: S1411S)
3. Agarose LE, quick dissolve (Apex BioResearch Products, catalog number: 20-102QD)
4. Bromophenol blue (Bio-Rad, catalog number: 1610404)
5. Chloroform, ethanol stabilized (Millipore Sigma, catalog number: 67-66-3)
6. 10× CutSmart buffer (New England Biolabs, catalog number: B7204S)
7. Cycloheximide (CHX) (Sigma, catalog number: C1988)
8. Dimethyl sulfoxide (DMSO) (Millipore Sigma, catalog number: MX1458-3)
9. Dithiothreitol (DTT) (Thermo Scientific, catalog number: 20290)
10. DNA Clean & Concentrator-25 kit (Zymo Research, catalog number: 11-305C)
11. DNase I (Rnase-free) (New England Biolabs, catalog number: M0303L)
12. *E. coli* Poly(A) polymerase (New England Biolabs, catalog number: M0276L)
13. 500 mM EDTA, pH 8.0 ULTROL grade (Millipore, catalog number: 324504)
14. Ethanol, 200 proof (Decon Laboratories, Inc., catalog number: 64-17-5)
15. 10 mg/mL ethidium bromide (Thermo Scientific, catalog number: 15585011)
16. Flexi Rabbit Reticulocyte Lysate System (Promega, catalog number: L4540)
17. 37% (w/v) formaldehyde (Fisher Chemical, catalog number: F79-500)
18. Glycerol, biotechnology grade (Amresco, catalog number: 0854-4L)
19. Glycogen, molecular biology grade (Thermo Scientific, catalog number: R0561)
20. Hi-Di Formamide (Thermo Scientific, Applied Biosystems, catalog number: 4401457)
21. HiScribe T7 High Yield RNA Synthesis kit (New England Biolabs, catalog number: E2040S)
22. iScript Reverse Transcription Supermix (Bio-Rad, catalog number: 1708841)
23. iTaq Universal SYBR Green Supermix, 5 mL (Bio-Rad, catalog number: 1725124)
24. 2 M KCl, pH 7.4 (MOLTOX, catalog number: 26-516.5)
25. 1 M MgCl_2_ (Thermo Scientific, catalog number: AM9530G)
26. Millennium RNA size marker (Thermo Scientific, catalog number: AM7150)
27. 10× MOPS buffer (KD Medical, catalog number: RGF-6170)
28. Nuclease-free water (Thermo Scientific, catalog number: AM9937)
29. Puromycin dihydrochloride (Sigma, catalog number: P8833)
30. 6× purple gel loading dye (New England Biolabs, catalog number: B7024A)
31. Quick-Load Purple 1 kb Plus DNA ladder (New England Biolabs, catalog number: N0550S)
32. RNA Clean & Concentrator-25 kit (Zymo Research, catalog number: 11-353B)
33. Rnase inhibitor, murine (New England Biolabs, catalog number: M0314L)
34. Sucrose, Ultra-pure, Rnase- & Dnase-free (VWR, catalog number: 57-50-1)
35. 10× TBE buffer (IBI Scientific, catalog number: IB70154)
36. 1 M Tris-HCl, pH 7.4 (Apex BioResearch Products, catalog number: 18-189)
37. TRIzol reagent (Ambion, catalog number: 15596018)
38. Xylene cyanol FF (Bio-Rad, catalog number: 1610423)
39. pcDNA3.1(+)/nLuc-3XFLAG (Addgene, catalog number: 127299)
40. Dual promoter plasmid pCR II (Thermo Scientific, catalog number: K207040)
41. pGL4.13 (Promega, catalog number: E6681)

**Solutions**

1. 100 mg/mL cycloheximide (CHX) (see Recipes)
2. 10 mg/mL CHX (see Recipes)
3. 5 mg/mL CHX (see Recipes)
4. 1 M DTT, Cleland’s Reagent (see Recipes)
5. 70% (v/v) ethanol (see Recipes)
6. 1 mg/mL ethidium bromide (see Recipes)
7. 10 mM GTP (see Recipes)
8. 1× MOPS buffer (see Recipes)
9. 10 mg/mL (~18 mM) puromycin (see Recipes)
10. 0.6 mM puromycin (see Recipes)
11. 2× ribosome dilution buffer (see Recipes)
12. RNA loading dye (see Recipes)
13. RNA sample buffer (see Recipes)
14. 60% (w/v) sucrose (see Recipes)
15. 35% (w/v) sucrose, buffered (see Recipes)
16. 1× TBE buffer (see Recipes)

**Recipes**

1. **100 mg/mL cycloheximide (CHX) (store at -20 °C)**
  **Table:** 

Reagent	Final concentration	Quantity
CHX	100 mg/mL	1 g
DMSO	n/a	To 10 mL
Total	n/a	10 mL
2. **10 mg/mL CHX (working solution, aliquot into single-use vials and store at -20 °C)**
  **Table:** 

Reagent	Final concentration	Quantity
100 mg/mL CHX	10 mg/mL	1 mL
Milli-Q water	n/a	9 mL
Total	n/a	10 mL
3. **5 mg/mL CHX (working solution, aliquot into single-use vials and store at -20 °C)**
  **Table:** 

Reagent	Final concentration	Quantity
10 mg/mL CHX	5 mg/mL	3 mL
Milli-Q water	n/a	3 mL
Total	n/a	6 mL
4. **1 M DTT, Cleland’s Reagent (store at -20 °C)**
  **Table:** 

Reagent	Final concentration	Quantity
DTT	1 M	1.542 g
Milli-Q water	n/a	To 10 mL
Total	n/a	10 mL
5. **70% (v/v) ethanol**
  **Table:** 

Reagent	Final concentration	Quantity
100% Ethanol	70% (v/v)	35 mL
Milli-Q water	n/a	15 mL
Total	n/a	50 mL
6. **1 mg/mL ethidium bromide**
  **Table:** 

Reagent	Final concentration	Quantity
10 mg/mL ethidium bromide	1 mg/mL	100 μL
Milli-Q water	n/a	900 μL
Total	n/a	1 mL
7. **10 mM GTP (store at -20 °C)**
  **Table:** 

Reagent	Final concentration	Quantity
100 mM GTP (from HiScribe T7 High Yield RNA Synthesis kit)	10 mM	50 μL
Nuclease-free water	n/a	450 μL
Total	n/a	500 μL
8. **1× MOPS buffer**
  **Table:** 

Reagent	Final concentration	Quantity
10× MOPS buffer	1×	60 mL
Milli-Q water	n/a	540 mL
Total	n/a	600 mL
9. **10 mg/mL (~18 mM) puromycin (store at -20 °C)**
  **Table:** 

Reagent	Final concentration	Quantity
Puromycin	10 mg/mL	1 g
Milli-Q water	n/a	To 100 mL
Total	n/a	100 mL
10. **0.6 mM puromycin (store at -20 °C)**
  **Table:** 

Reagent	Final concentration	Quantity
18 mM puromycin	0.6 mM	66.67 μL
Milli-Q water	n/a	1.933 mL
Total	n/a	2 mL
11. **2× ribosome dilution buffer (store at 4 °C)**
  **Table:** 

Reagent	Final concentration	Quantity
1 M Tris-HCl, pH 7.4	40 mM	2 mL
2 M KCl	280 mM	7 mL
1 M MgCl_2_	20 mM	1 mL
100 mg/mL CHX	0.2 mg/mL	100 μL *Add day of use
1 M DTT	2 mM	100 μL *Add day of use
Milli-Q water	n/a	39.8 mL
Total	n/a	50 mL
12. **RNA loading dye**
  **Table:** 

Reagent	Final concentration	Quantity
Glycerol	50% (v/v)	5 mL
500 mM EDTA, pH 8.0	100 mM	2 mL
Bromophenol blue	2.5 mg/mL	25 mg
Xylene cyanol FF	2.5 mg/mL	25 mg
Milli-Q water	n/a	3 mL
Total	n/a	10 mL
13. **RNA sample buffer (make fresh day of use)**
  **Table:** 

Reagent	Final concentration	Quantity
37% (w/v) formaldehyde	9% (w/v)	35 μL
Hi-Di formamide	69% (v/v)	100 μL
10× MOPS buffer	0.7×	10 μL
Total	n/a	145 μL
14. **60% (w/v) sucrose**
  **Table:** 

Reagent	Final concentration	Quantity
Sucrose	60% (w/v)	300 g
Milli-Q water	n/a	To 500 mL
Total	n/a	500 mL
15. **35% (w/v) sucrose, buffered (store at 4 °C)**
  **Table:** 

Reagent	Final concentration	Quantity
60% sucrose (w/v)	35% (w/v)	29.167 mL
1 M Tris-HCl, pH 7.4	20 mM	1 mL
2 M KCl	140 mM	3.5 mL
1 M MgCl_2_	10 mM	0.5 mL
100 mg/mL CHX	0.1 mg/mL	50 μL *Add day of use
1M DTT	1 mM	50 μL *Add day of use
Milli-Q water	n/a	15.733 mL
Total	n/a	50 mL
16. **1× TBE buffer**
  **Table:** 

Reagent	Final concentration	Quantity
10× TBE buffer	1×	100 mL
Milli-Q water	n/a	900 mL
Total	n/a	1 L

**Laboratory supplies**

1. 0.2 mL, open-top thick wall polycarbonate tube, 7 mm × 20 mm (Beckman Coulter, catalog number: 343775)
2. 1.7 mL microcentrifuge tube, clear (Olympus Plastics, catalog number: 24-282)
3. 500 mL Erlenmeyer flask
4. 8-strip PCR tubes (Olympus Plastics, catalog number: 27-125UA)
5. Hard-shell PCR plates, 96-well, thin-well (Bio-Rad, catalog number: HSP9601)
6. Ice
7. Ice buckets
8. Light-dry tissue wipes (VWR, catalog number: 82003-820)
9. Microseal ‘B’ seals (Bio-Rad, catalog number: MSB1001)
10. Nitrile gloves
11. P10, P20, P200, and P1000 calibrated pipettes
12. P10, P20, P200, and P1000 pipette tips (VWR, catalog numbers: 76323-388, 76323-390, and 76323-456)
13. Parafilm
14. Plastic wrap
15. Tube racks

## Equipment

-80 °C freezer
-20 °C freezer
3. 4 °C refrigerator
4. Aspirator
5. CFX Connect Real-Time System (Bio-Rad, catalog number: 1855201)
6. Eppendorf centrifuge 5430 (Eppendorf, catalog number: 022620509)
7. Fume hood
8. GelDoc Go Gel imaging system with Image Lab Touch software (Bio-Rad, catalog number: 12009077)
9. Microwave
10. MilliporeSigma Synergy ultrapure water purification system (Fischer Scientific, catalog number: SYNS0HFUS)
11. NanoDrop One Microvolume UV-Vis spectrophotometer (Thermo Scientific, catalog number: ND-ONE-W)
12. OWL EasyCast B1 Mini Gel electrophoresis system (Thermo Scientific, catalog number: B1-BP)
13. OWL EasyCast B1A Mini Gel electrophoresis system (Thermo Scientific, catalog number: 09-528-110)
14. Paper towels
15. Plate centrifuge, PerfectSpin P (VWR Peqlab, catalog number: PEQL91-PSPIN-P)
16. Pointed tweezers
17. S100-AT3 fixed angle rotor (Thermo Scientific, catalog number: 45585)
18. Sorvall Discovery M120 SE micro-ultracentrifuge (Hitachi)
19. T100 thermal cycler (Bio-Rad, catalog number: 1861096)
20. Vortex mixer (Benchmark, catalog number: BV1000)

## Software and datasets

1. Bio-Rad CFX Maestro
2. Microsoft Excel
3. Prism GraphPad Molarity Calculator (https://www.graphpad.com/quickcalcs/molarityform/)
4. Oligo Calc: Oligonucleotide Properties Calculator (http://biotools.nubic.northwestern.edu/OligoCalc.html)
5. Transcription and Translation Tool (https://biomodel.uah.es/en/lab/cybertory/analysis/trans.htm)

## Procedure

- **In vitro transcription of ARCA-capped reporter mRNAs**
  1. Linearize normalizing control and experimental reporter plasmids by restriction enzyme digestion. Separate reporters can be linearized in parallel.
*Notes:*
*i. For the normalizing control reporter, we typically use FireFly Luciferase (FFLuc) subcloned from pGL4.13 into pCR II, which is linearized with HindIII. For the experimental reporter, we typically use nanoLuciferase (nLuc) subcloned from pNL1.1 into pcDNA3.1(+), which is linearized with either XbaI or PspOMI. Both plasmids are available upon request from the corresponding author; pcDNA3.1(+)/nLuc-3XFLAG is also available from Addgene.*
*ii. Choose a restriction endonuclease that cleaves downstream of the reporter coding sequence and either produces blunt ends or 5 overhangs. Restriction endonucleases that produce 3′ overhangs should be avoided as they can elicit aberrant and antisense transcription (Schenborn and Mierendorf, 1985). Choose a restriction enzyme that will produce the desired 3′ untranslated region (UTR). For example, linearizing pcDNA3.1(+)/nLuc via either XbaI or PspOMI produces a short (10 base or 16 base, respectively) 3′ UTR that works very well in our hands. We have not tested if shorter 3′ UTRs affect translation efficiency of reporter mRNAs in this system, nor have we tested if the length of the 3′ UTR affects the sensitivity of this protocol. In general, 3′ UTRs typically contain inhibitory and stimulatory translational control elements; thus, smaller 3′ UTRs will contain fewer unexpected translational control elements.*
    - In a microcentrifuge tube, combine 100 μL of plasmid DNA (300 ng/μL), 20 μL of 10× CutSmart buffer, 20 μL of restriction enzyme (400–800 U total), and 60 μL of Milli-Q water. Gently mix by inversion 20 times and collect contents by a short centrifugation spin (e.g., 2–3 s in a minicentrifuge or microcentrifuge).
    - Digest the plasmid for 4 h at 37 °C and then hold at 4 °C. **Pause point:** Store completed digest at -20 °C.
*Note: Despite the excess of restriction enzyme in the reaction, we suggest a 4 h incubation for complete linearization due to the relatively large massof plasmid DNA.*
    - During the restriction digest, cast a 0.8% (w/v) agarose gel in an OWL EasyCast B1A Mini Gel electrophoresis system with a 1.5 mm 10-well comb. Add 100 mL of 1× TBE buffer with 0.8 g of agarose in a 500 mL Erlenmeyer flask and then loosely plug the flask with a folded-up paper towel. Heat in the microwave for 1.5 min or until the agarose is completely dissolved. Allow to cool for 10 min on the benchtop and then add 5 μL of 10 mg/mL ethidium bromide. Gently swirl to mix and let the flask cool on the benchtop until it can be held comfortably for 10 s (or place in a 65 °C water or bead bath for 30 min). Pour ~70 mL into the casting tray and let the gel solidify at room temperature for ~1 h. Remove combs by gently pulling them up vertically. Remove and rotate the casting tray so that the wells are near the cathode. Fill the gel tank and completely cover the agarose gel with 1× TBE buffer (~350 mL total).
  2. Purify linearized plasmids using the DNA Clean and Concentrator-25 kit and the supplied solutions.
    - Perform all steps at room temperature and centrifugation at 16,000× *g* for 30 s, unless specified. To the 200 μL digest, add 800 μL of DNA binding buffer and mix by gentle inversion 20 times. Transfer mixture to the Zymo-spin column in a collection tube. Centrifuge and discard flowthrough. Wash the column with 200 μL of DNA wash buffer by centrifugation. Discard flowthrough and repeat wash step. Transfer column into a fresh and pre-labeled 1.7 mL microcentrifuge tube. Add 30 μL of nuclease-free water directly to the column (the white resin at the bottom of the column) and incubate at room temperature for 1 min. Elute by centrifugation (1 min, 16,000× *g*, room temperature). Store on ice for immediate use or at -20 °C for long-term storage.
    - Determine DNA concentration and purity by UV spectrophotometry (e.g., NanoDrop).
*Note: The Zymo-spin IICR column has a reported capacity of 25 μg of DNA. This protocol calls for 30 μg of DNA, which allows us to max out the column, resulting in most reporter plasmids eluting at ~1,000 ng/μL.*
    - Confirm linear plasmid integrity by 0.8% (w/v) agarose gel electrophoresis. Add 5 μL of Quick-Load Purple 1 kb Plus DNA ladder to the first well. On a piece of parafilm or in a microcentrifuge tube, gently mix 1 μL of purified linear plasmid DNA (250–500 ng/μL), 1 μL of purple gel loading dye (6×), and 4 μL of Milli-Q water, and then load the entire sample into a single well. Run the agarose gel at 120 V (constant) for 30–45 min or until the desired resolution. Image gel via UV transillumination to confirm a single band that is running at the expected molecular weight.
*Note: If using two combs per casting tray, use the bottom half of the agarose gel before using the top half, as ethidium bromide in the gel runs toward the cathode. Unused parts of the gel may be stored for up to one week in plastic wrap at 4 °C.*
  3. Synthesize in vitro–transcribed reporter mRNA using the HiScribe T7 High Yield RNA Synthesis kit.
    - To resuspend the ARCA cap analog to 40 mM, first quickly collect the contents (1 μmol) by a short centrifugation and then add 25 μL of nuclease-free water to the pellet. Gently mix by flicking the tube and collect the contents by brief centrifugation. Repeat the gently mixing procedure for a total of five times.
    - For a typical 10 μL reaction, combine on ice 1 μL of linear reporter plasmid (500 ng/μL), 0.5 μL of RNase inhibitor, 1 μL of 10× T7 reaction buffer, 1 μL of 100 mM ATP, 1 μL of 100 mM UTP, 1 μL of 100 mM CTP, 1 μL of 10 mM GTP, 2 μL of 40 mM ARCA cap analog, 1 μL of T7 RNA polymerase mix, and 0.5 μL of nuclease-free water in a PCR tube or microcentrifuge tube. **It is critical to use 1 μL of 10 mM GTP and not 1 μL of 100 mM GTP.** The 8:1 ARCA cap analog to GTP ratio ensures 90% co-transcriptional capping efficiency (Krieg and Melton, 1987).
*Notes:*
*i. If transcribing multiple mRNAs at one time, assemble a master mix of all components except linear plasmid templates. Use 9 μL of master mix with 1 μL of linear plasmid template (~500 ng/μL). Reactions can also be scaled up linearly to 20 μL but beware of exceeding the binding capacity of the RNA clean up columns (see below).*
*ii. Most T7 RNA polymerase–mediated mRNA synthesis kits with a cap analog pre-mixed with the NTPs use a 4:1 ARCA cap to GTP ratio, which only provides ~80% co-transcriptional capping efficiency. These cap analog pre-mixed kits also do not provide the ability to generate A-capped or non-methylated G-capped mRNAs to test cap-dependency.*
    - Perform in vitro transcription for 2 h at 30 °C using a thermal cycler (for PCR tube) or heat block (for microcentrifuge tube).
*Note: In our hands, transcription at 30 °C yielded purer mRNA than transcription at 37 °C.*
    - To remove the DNA template, add 1 μL of DNase I (RNase-free) to each 10 μL reaction and gently mix by inversion. Incubate at 37 °C for 15 min.
    - If polyadenylation of the RNA is desired, combine and gently mix on ice the 11 μL DNase-treated capped and transcribed mRNA reaction with 5 μL of 10× Poly(A) polymerase buffer, 5 μL of 10 mM ATP, 1 μL of *E. coli* Poly(A) polymerase, and 28 μL of nuclease-free water, followed by incubation at 37 °C for 1 h.
      *Note: This protocol uses the* ***nuclease-treated*** *Flexi rabbit reticulocyte lysate (RRL) system from Promega. It is not entirely necessary to polyadenylate in vitro–transcribed mRNA for efficient translation using* ***nuclease-treated*** *RRL. Polyadenylation provides a less than two-fold enhancement of reporter mRNA translation in* ***nuclease-treated*** *RRL (Soto Rifo, 2007). If polyadenylation is skipped, add 39 μL of nuclease-free water to bring the sample volume to 50 μL. It is worth noting that the described cDNA synthesis reagents below use both random hexamers and oligo-dT primers. If using different reagents that only contain oligo-dT primers for cDNA synthesis, polyadenylation is required for detection of pelleted, ribosome-bound mRNA.*
    - If using a PCR tube, transfer the entire contents to a microcentrifuge tube.
  4. Purify mRNA using the RNA Clean and Concentrator-25 kit and the supplied solutions.
    - Perform all steps at room temperature and centrifugation at 16,000× *g* for 1 min, unless specified. To each 50 μL of mRNA sample, add 100 μL of RNA binding buffer and gently mix by inversion 20 times. Add 150 μL of 100% ethanol to each reaction and gently mix by inversion 20 times. Transfer the entire sample to the Zymo-Spin IICR Column in a collection tube and centrifuge. Discard the flowthrough. Add 400 μL of RNA prep buffer to the column, centrifuge, discard flowthrough, and place column back into the collection tube. Add 700 μL of RNA wash buffer to the column, centrifuge, discard flowthrough, and place column back into the collection tube. Add 400 μL of RNA wash buffer to the column, centrifuge for 4 min, discard flowthrough, and place column into a new RNase-free 1.7 mL microcentrifuge tube. Add 75 μL of nuclease-free water directly to the column matrix and allow to incubate at room temperature for 1 min. Elute by centrifugation. Store mRNA on ice moving forward.
    - Determine RNA concentration and purity by UV spectrophotometry (e.g., NanoDrop).
    - Aliquot mRNA in 3 μL volumes in PCR strips and store at -80 °C. **Pause point.**
  5. Confirm in vitro–transcribed mRNA quality and purity by denaturing agarose gel electrophoresis.
    - Cast a denaturing 0.8%–1% (w/v) agarose formaldehyde gel in OWL EasyCast B1 Mini Gel electrophoresis system with a 1.5 mm 10-well comb placed in the top comb slot. Add 0.8–1 g of agarose to 80 mL of Milli-Q water in a 500 mL Erlenmeyer flask loosely plugged with a folded-up paper towel. Microwave for 1.5 min or until the agarose is dissolved and gently swirl to mix. Cool on countertop for 2 min.
    - In a fume hood, add 10 mL of 10× MOPS buffer and 10 mL of 37% formaldehyde to the flask containing dissolved agarose. Gently swirl to mix and allow to cool for 5–10 min.
    - Pour 100 mL into the casting tray and let the gel solidify at room temperature for ~1 h in the dark by loosely covering with aluminum foil.
    - Prepare mRNA samples by mixing 500 ng of in vitro–transcribed mRNA, 1 μL of 1 mg/mL ethidium bromide, 1 μL of RNA loading dye (see Recipes), 5 μL of freshly-prepared RNA sample buffer (see Recipes), and nuclease-free water to 15 μL total volume.
    - Prepare the RNA ladder by mixing 1 μL of Millennium RNA size marker (1 μg/μL), 1 μL of 1 mg/mL ethidium bromide, 1 μL of RNA loading dye (see Recipes), 3 μL of RNA sample buffer (see Recipes), and 4 μL of nuclease-free water.
    - Heat ladder and samples at 70 °C for 5 min; then, place the ladder and samples on ice for 2 min.
    - Remove combs by gently pulling up vertically. Remove and rotate the casting tray so that the wells are near the cathode. Fill the gel tank and completely cover the agarose gel with 1× MOPS buffer (600 mL total, see Recipes).
    - Load ladder and samples on the gel.
    - Run at 60 V (constant) (or 5–10 V/cm gel width) in the dark (covered with aluminum foil) for 3–4 h (when the dye front reaches 2 cm from the bottom of the gel) or until the desired resolution.
    - Image gel via UV transillumination to confirm a single band is running at the expected molecular weight (polyadenylating mRNA will add to the expected molecular weight).
*Note: Non-polyadenylated in vitro–transcribed mRNA will run as a single, crisp band at the expected molecular weight on a denaturing agarose gel. The same in vitro–transcribed mRNA that is polyadenylated will appear ~100–200 nt heavier as a slightly broader band. An additional control reaction lacking the Poly(A) polymerase can be included to better define the poly(A) tail length. Large smears spanning far beyond the expected molecular weight indicate RNA degradation, rolling circle transcription due to the presence of non-linear template DNA, nondenatured RNA secondary structure, or errors during RNA clean up steps. We have also found that making fresh, day-of-use RNA sample buffer is critical.*
- **In vitro translation**
  1. Pre-cool the Sorvall Discovery M120 SE micro-ultracentrifuge and S100-AT3 rotor to 4 °C prior to setting up in vitro translation reactions. The S100-AT3 rotor can hold up to 20 samples.
*Note: For each experimental mRNA, at least six samples should be prepared and translated: three replicates that are translated and not puromycin-treated and three replicates that are translated and puromycin-treated (step B3). Each of the six samples is mixed with a translated normalizing control mRNA-containing reaction (step B2), then diluted and layered on top of individual sucrose cushions (step C1).*
  2. In vitro translation of normalizing control mRNA:
    - Dilute normalizing mRNA to 15 fmol/μL in nuclease-free water and keep on ice. To do so, use the plasmid DNA sequence from the first transcribed nucleotide of the T7 RNA polymerase promoter to the restriction endonuclease cut site and determine the corresponding RNA sequence using the online Transcription and Translation Tool (see Software). Then, calculate the molecular weight of the control mRNA reporter with the online Oligo Calc: Oligonucleotide Properties Calculator (**be sure to use the ssRNA setting**; see Software). Using its molecular weight from above and the online Prism GraphPad Molarity Calculator (see Software), calculate the mass required for 3 nM in 10 μL. This is the mass of mRNA required in the complete 10 μL in vitro translation reaction for 3 nM mRNA (final). Dilute the purified mRNA in nuclease-free water such that 2 μL of RNA contains the mass calculated above, resulting in a final concentration of 15 fmol/μL. Other similar online tools are available and would suffice.
    - On ice in a PCR tube, set up the following 10 μL translation reaction: mix 2 μL of 15 fmol/μL in vitro–transcribed mRNA, 3 μL of Flexi RRL (nuclease-treated), 0.1 μL of 1 mM amino acid mixture minus leucine, 0.1 μL of amino acid mixture minus methionine, 0.2 μL of 25 mM Mg(OAc), 0.4 μL of 2.5 M KCl, 0.2 μL of RNase inhibitor, and 4 μL of nuclease-free water. **If performing multiple reactions, prepare a master mix of all components and split into 10 μL reactions.**
    - Perform in vitro translation for 15 min at 30 °C in a thermal cycler.
    - Immediately place samples on ice.
    - To each sample, add 4 μL of 5 mg/mL CHX (see Recipes) (final 1.43 mg/mL). Gently mix by inversion 20 times and collect contents by a short centrifugation spin. Samples should now be 14 μL. Keep samples on ice until step C1.
*Note: Cycloheximide is added in step B2 to robustly inhibit elongation and preserve ribosome-bound mRNAs when added to experimental samples that contain puromycin in step B3.*
  3. In vitro translation of experimental mRNA:
    - Dilute experimental mRNA(s) to 15 fmol/μL (as in step B2a) in nuclease-free water and keep on ice.
    - On ice in a PCR tube, set up the following 10 μL translation reaction: mix 2 μL of 15 fmol/μL in vitro transcribed mRNA, 3 μL of Flexi RRL (nuclease-treated), 0.1 μL of 1 mM amino acid mixture minus leucine, 0.1 μL of amino acid mixture minus methionine, 0.2 μL of 25 mM Mg(OAc), 0.4 μL of 2.5 M KCl, 0.2 μL of RNase inhibitor, and 4 μL of nuclease-free water. If translating multiple experimental reporters or many biological replicates, prepare a master mix with all components except for the mRNA; add 8 μL of the master mix to 2 μL of 15 fmol/μL in vitro–transcribed mRNA.
    - Perform in vitro translation for 15 min at 30 °C in a thermal cycler.
    - Immediately place samples on ice.
    - To each sample, add 2 μL of nuclease-free water or 0.6 mM puromycin (see Recipes) (final 0.1 mM). Gently mix by inversion 20 times and collect contents by a short centrifugation spin.
    - Place puromycin-containing samples on a thermal cycler and incubate at 30 °C for 30 min. Keep samples without puromycin (i.e., samples with water added) on ice.
    - Place all samples on ice and immediately add 2 μL of 10 mg/mL CHX (see Recipes) (final 1.43 mg/mL). Gently mix by inversion 20 times and collect contents by a short centrifugation spin. Keep samples on ice.
    - All translation reactions should now be 14 μL.
- **Ribosomal pelleting through low-speed sucrose cushion**
  1. Prepare samples for low-speed ribosomal pelleting through a sucrose cushion.
    - Mix a 14 μL normalizing control mRNA-containing reaction tube and a 14 μL experimental mRNA-containing reaction tube for a total sample volume of 28 μL. Keep samples on ice.
    - Add 28 μL of ice-cold 2× ribosome dilution buffer (that contains freshly added CHX and DTT, see Recipes). Each sample should now be 56 μL.
    - Gently mix by inversion 20 times and collect contents by a short centrifugation spin. Keep samples on ice.
  2. Prepare sucrose cushions and overlay samples.
    - Label 7 mm × 20 mm polycarbonate thick-walled tubes. Mark a single spot on the rim of each tube (Figure 2). Place tubes on a homemade ice bucket made from a P1000 tip box that fits the 7 mm × 20 mm tubes (Figure 3).
      **Figure 2.**
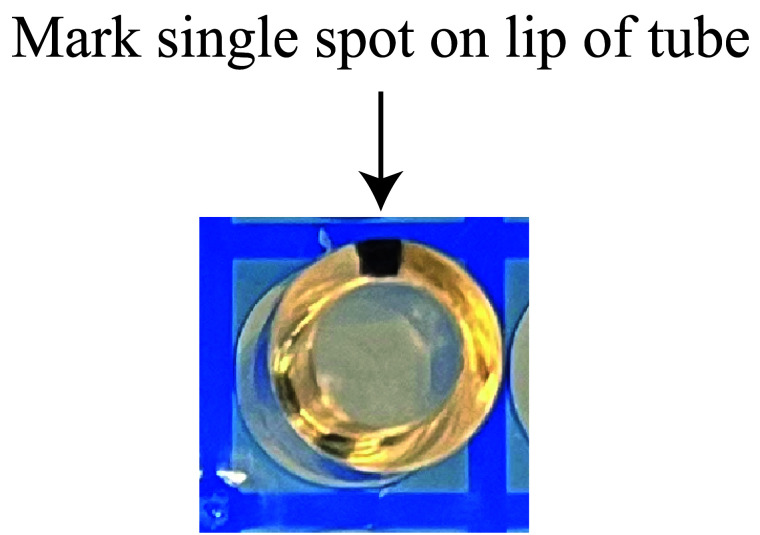
Example of how to mark the 7 mm × 20 mm tube to predict the side on which the pellet will form
      **Figure 3.**
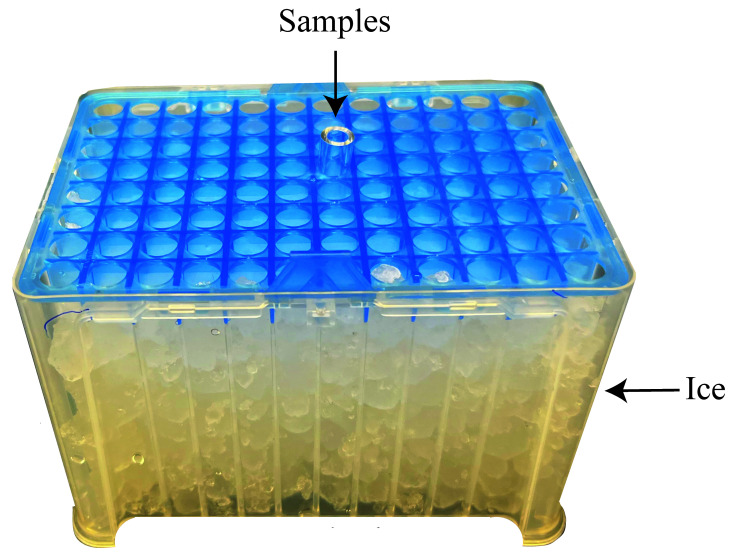
Example of homemade ice bucket using a rechargeable P1000 tip box to hold 7 mm × 20 mm thick-walled tubes
    - Add 130 μL of ice-cold 35% buffered sucrose (that contains freshly added CHX and DTT, see Recipes) to the bottom of each tube.
    - Very carefully, overlay all 56 μL of the diluted sample from step C1 on top of the sucrose cushion.
  3. Low-speed centrifugation
    - Without disturbing the sample–sucrose interface, place the tubes into the pre-chilled S100-AT3 rotor. Be sure to position the marked spot facing outward and toward the back of the rotor. This spot will indicate the side of the tube where the ribosome pellet will be located after centrifugation (Figure 4). Using pointed tweezers for this step is helpful.
      **Figure 4.**
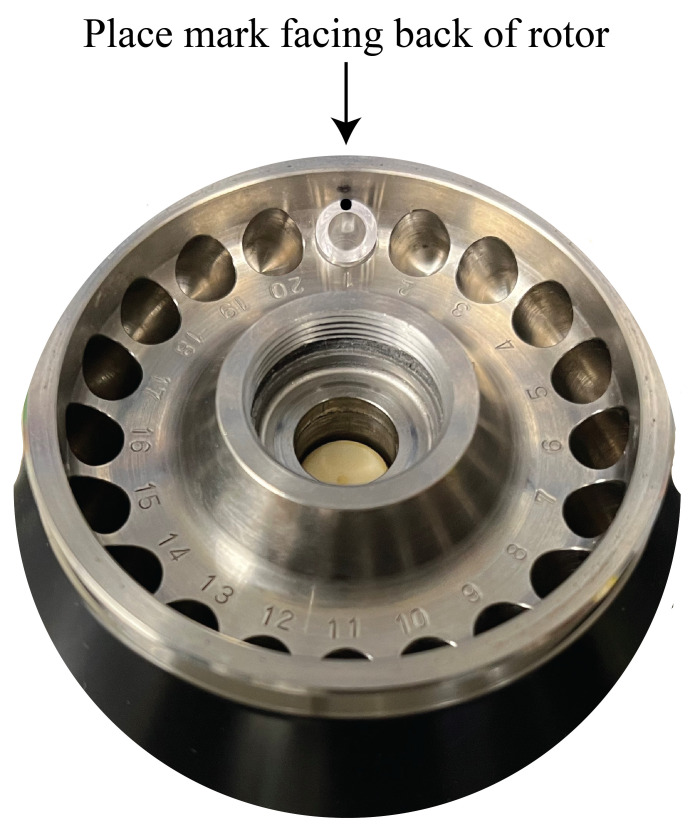
Example of how to orient the 7 mm × 20 mm tube in the S100-AT3 rotor
    - Carefully place the rotor into the pre-cooled Sorvall Discovery M120 SE micro-ultracentrifuge.
    - Centrifuge samples at 50,000× *g* for 1 h at 4 °C. Use an acceleration setting of 9 (fastest setting) and a deceleration setting of 5 (middle setting).
    - Carefully, remove the tubes from the rotor and place them on ice using the homemade ice bucket for the 7 mm × 20 mm tubes. Using pointed tweezers for this step is helpful.
    - Using a pipettor, remove and discard the supernatant without disturbing the pellet. The glossy clear pellet should be at the bottom edge of the tube below the mark that was facing outward and toward the back of the rotor during centrifugation.
    - Resuspend the pellet in TRIzol.
      i. First, add 200 μL of TRIzol to a new, labeled, nuclease-free microcentrifuge tube.
      ii. Add 100 μL of TRIzol to the ribosome pellet in the 7 mm × 20 mm tube and mix 20 times by gently pipetting up and down. The pellet will dissociate from the tube wall when TRIzol is first added and will float in solution until it ultimately dissolves.
      iii. Transfer this 100 μL to the microcentrifuge tube (now containing 300 μL).
      iv. Add another 200 μL of TRIzol to the 7 mm × 20 mm tube to wash off any remaining ribosomes by gently pipetting up and down 10 times.
      v. Transfer the 200 μL sample to the labeled microcentrifuge tube (now containing 500 μL total)
    - Mix samples end-over-end at room temperature for 15 min at 15 rpm. Collect contents by a short centrifugation spin. **Pause point:** Store samples at -80 °C.
- **RNA extractions, cDNA synthesis, and RT-qPCR**
  1. RNA extractions
    - Thaw samples at room temperature if necessary. Add 100 μL of chloroform to each sample.
    - Mix vigorously by hand for 1 min (do not vortex).
    - Centrifuge for 15 min at 12,000× *g* at 4 °C.
    - Without disturbing or touching the protein interface, carefully remove the top 200 μL clear aqueous layer and transfer it to a new, labeled, nuclease-free microcentrifuge tube.
    - Add 1.5 μL glycogen (20 mg/mL) to each sample. Upon dispensing, wash the tip in the aqueous phase by gently pipetting up and down three times.
    - To each sample, add 500 μL of 100% isopropanol. Gently mix by inversion 20 times.
    - Centrifuge for 15 min at 12,000× *g* at 4 °C.
*Note: When pelleting RNA in microcentrifuge tubes, place the hinge upright facing the outside of the rotor. This will allow you to predict the location of the pellet at the bottom of the tube on the same side of the hinge.*
    - Aspirate off the isopropanol until ~100 μL remains in the tube, leaving the RNA pellet untouched. Remove the final ~100 μL with a P200 pipette. The pellet should be white but very small.
    - Add 600 μL of ice-cold 70% ethanol. Vortex each sample for 1 s.
    - Centrifuge for 15 min at 12,000× *g* at 4 °C.
    - Aspirate off the ethanol until ~100 μL remains in the tube, leaving the RNA pellet untouched. Remove the final ~100 μL with a P200 pipette and finally a P10 pipette. Aspirate any remaining ethanol off the tube walls. Allow to air dry with the top open for 2–3 min at room temperature on the bench.
    - Place tubes on ice, add 30 μL of nuclease-free water to the pellet, and let stand on ice for 2 min. Gently resuspend the pellet by pipetting up and down 20 times. Be sure to wash down the side of the tube (same side as the hinge) to ensure complete resuspension of the RNA pellet.
  2. cDNA synthesis
    - On ice, combine 16 μL of RNA and 4 μL of 5× iScript Reverse Transcription Supermix. Mix by gentle inversion 20 times and collect the contents by a short centrifugation spin. Store the remaining 14 μL of RNA at -80 °C.
    - Using a thermal cycler, reverse transcribe using:
      i. 25 °C for 5 min (priming)
      ii. 46 °C for 20 min (reverse transcription)
      iii. 95 °C for 1 min (reverse transcriptase inactivation and RNA cleavage)
      iv. Hold at 4 °C
    - To each 20 μL cDNA sample, add 180 μL of nuclease-free water for a 1:10 dilution. Gently mix by inversion 20 times and collect contents by a short centrifugation spin. **Pause point:** Store samples at -20 °C.
  3. RT-qPCR
    - Design a 96-well plate layout scheme for all samples and negative controls. Each cDNA sample will be amplified with two primer sets: one targeting the normalizing control mRNA and the other targeting the experimental mRNA. A no-template negative control (where water is added instead of cDNA) should also be included for each primer set. Perform at least technical duplicates for each sample and primer set.
    - Dilute qPCR primers by mixing 510 μL of nuclease-free water, 45 μL of 10 μM forward primer, and 45 μL of 10 μM reverse primer. See General notes below for the primer sequences we used for FFLuc and nLuc.
    - Our typical RT-qPCR reaction is 15 μL. Each well will contain 7.5 μL of iTaq Universal SYBR Green Supermix (2×), 1.5 μL of the 1:10 diluted cDNA, and 6 μL of diluted primers. For both the normalizing control and experimental primer sets, create a master mix of iTaq Universal SYBR Green Supermix and diluted primers. Add 13.5 μL of this master mix to the appropriate wells. Then, carefully add 1.5 μL of the appropriate diluted cDNA samples. The no-template negative control contains 13.5 μL of appropriate master mix and 1.5 μL of nuclease-free water.
    - Seal the plate with a microseal ‘B’ seal. Be sure not to touch the top of the plastic directly, but rather apply pressure to the seal by using a clean tissue wipe (or Kimwipe). Remove and discard the perforated edges.
    - Centrifuge the plate in a plate centrifuge for ~30 s to pull the samples to the bottom of the wells.
    - Place the plate into the Bio-Rad CFX Connect Real-Time System.
    - Run the following RT-qPCR program:
      i. One cycle of 95 °C for 3 min.
      ii. Forty cycles of 95 °C for 10 s, 60 °C for 30 s, followed by a Plate Read.
      iii. Melt curve from 65 °C to 95 °C with an increment of 0.5 °C for 5 s and a Plate Read.
*Note: If using new primer sets, peel back the plastic film after a run and take out 10 μL to confirm the expected size amplicon on a 2% (w/v) agarose gel.*

## Data analysis

For each experimental mRNA, at least six samples should be prepared and translated: three replicates that are translated and not puromycin-treated and three replicates that are translated and puromycin-treated (step B3). Each of the six samples is mixed with a translated normalizing control mRNA reaction (step B2), then diluted and layered on top of individual sucrose cushions (step C1). During RT-qPCR, each cDNA should be assayed in at least technical duplicates. Primer sets for both the normalizing control mRNA and experimental mRNA should be used in separate wells on the same plate.

Using Bio-Rad CFX Maestro, select the Gene Expression window when the RT-qPCR run is complete. In the Experimental Settings tab, select the normalizing control mRNA as the Reference. This will normalize the signal of the experimental mRNA (i.e., nLuc) to the signal of the normalizing control mRNA (i.e., FFLuc) to account for any error during RNA extraction and/or cDNA synthesis. Once the gene expression has been calculated by the CFX Maestro software, export the data and perform the remaining analysis in Excel. For each experimental reporter, group the without puromycin-treatment replicates and set to 100%. Then, determine the relative signal for each replicate with puromycin-treatment. Statistical comparisons can be made using an unpaired *t*-test with Welch’s correction. For nLuc as the experimental mRNA reporter, we typically observe a relative ~60% reduction in signal with puromycin treatment (Scarpitti et al., 2022).

Two additional controls should be incorporated in step B3:

1) A no-template negative control, where water is added instead of either reporter mRNA, is critical to ensure specificity of RT-qPCR primers and determine background levels of detection.

2) To confirm that the low-speed sucrose cushions are working as expected to selectively pellet ribosome-bound mRNA, set up translation reactions with the experimental mRNA without puromycin, incubate for 15 min on ice instead of 30 °C (step B3c), and proceed as directed above. The ice-incubated sample should have very few, if any, ribosomes loaded on the experimental mRNA and should be minorly detected by RT-qPCR when compared to the 30 °C–incubated sample.

## Validation of protocol

This protocol was validated in Scarpitti et al. (2022) *Journal of Biological Chemistry*, DOI: 10.1016/j.jbc.2022.102660. See Figure 6E and 6F.

## General notes and troubleshooting

These in vitro translation reaction conditions have been optimized to be in the dynamic linear range for time and mRNA input for a range of reporter mRNAs (Kearse et al., 2016). These same conditions are also sufficient for A-capped Internal Ribosome Entry Site (IRES)-mediated reporter mRNAs. The final concentration of reagents in the in vitro translation reactions are 3 nM mRNA, 30% (v/v) RRL, 1 μM amino acid mixture minus leucine, 1 μM of amino acid mixture minus methionine, 0.5 mM Mg(OAc), 100 mM KCl, and 0.8 U/μL RNase inhibitor. Adjust the amount of KCl added to translation reactions if also including recombinant protein or protein synthesis inhibitors that are stored in KCl-containing buffers. The final KCl concentration should be kept constant at 100 mM. Altering the KCl concentrations will alter cap dependency. We have found that any amount of NaCl or LiCl is very inhibitory to in vitro translation using RRL.

We empirically optimized the 0.1 mM puromycin (final) for use with the in vitro translation reaction conditions described in this protocol. Increasing puromycin beyond 0.1 mM did not further reduce the amount of mRNA co-pelleted in a translation-dependent manner. We also optimized the 50,000× *g* sucrose cushion in the Sorvall Discovery M120 SE micro-ultracentrifuge and S100-AT3 rotor. Increased centrifugal force resulted in loss of specificity for pelleting ribosome-bound mRNAs in a translation-dependent manner. Both variables may have to be re-optimized if using different translation extracts, if making substantial alterations to the translation reaction conditions, and/or if using a different rotor.

If testing ribosome stalling by an mRNA-binding protein (Scarpitti et al., 2022), we recommend an additional control where the messenger ribonucleoprotein (mRNP) is formed but not translated (i.e., step B3c is skipped). Some mRNPs may be heavy enough to pellet alone without any bound ribosomes during the low-speed centrifugation. This control confirms that the mRNA detected in the pellet is translation- and ribosome-dependent. We have shown that FMRP forms an mRNP with nLuc mRNA and stalls ribosomes and that FMRP•nLuc mRNP alone does not pellet in the low-speed sucrose cushion unless translated. In the case of FMRP, a pre-incubation step with FMRP and mRNA was included prior to addition to the in vitro translation reaction (Scarpitti et al., 2022). However, this pre-incubation step may be unnecessary in some cases and should be optimized for each RNA-binding protein of interest.

Lastly, while we have always used FFLuc as the normalizing control mRNA and nLuc as the experimental mRNA, we believe other reporters (e.g., GFP, mCherry, Renilla Luciferase) would function just as well for either the normalizing control or experimental mRNA. We have previously cloned FFLuc from pGL4.13 into the dual promoter plasmid pCR II. The use of pCR II is not required, but pCR II/FFLuc under control of the T7 RNA polymerase promoter is available upon request. pcDNA3.1(+)/nLuc-3XFLAG, which contains a T7 RNA polymerase promoter upstream of nLuc, is available upon request and from Addgene.

See below for reporter coding sequences and qPCR primer sequences.

**FireFly Luciferase (FFLuc; from pGL4.13)**

ATGGAAGATGCCAAAAACATTAAGAAGGGCCCAGCGCCATTCTACCCACTCGAAGACGGGACCGCCGGCGAGCAGCTGCACAAAGCCATGAAGCGCTACGCCCTGGTGCCCGGCACCATCGCCTTTACCGACGCACATATCGAGGTGGACATTACCTACGCCGAGTACTTCGAGATGAGCGTTCGGCTGGCAGAAGCTATGAAGCGCTATGGGCTGAATACAAACCATCGGATCGTGGTGTGCAGCGAGAATAGCTTGCAGTTCTTCATGCCCGTGTTGGGTGCCCTGTTCATCGGTGTGGCTGTGGCCCCAGCTAACGACATCTACAACGAGCGCGAGCTGCTGAACAGCATGGGCATCAGCCAGCCCACCGTCGTATTCGTGAGCAAGAAAGGGCTGCAAAAGATCCTCAACGTGCAAAAGAAGCTACCGATCATACAAAAGATCATCATCATGGATAGCAAGACCGACTACCAGGGCTTCCAAAGCATGTACACCTTCGTGACTTCCCATTTGCCACCCGGCTTCAACGAGTACGACTTCGTGCCCGAGAGCTTCGACCGGGACAAAACCATCGCCCTGATCATGAACAGTAGTGGCAGTACCGGATTGCCCAAGGGCGTAGCCCTACCGCACCGCACCGCTTGTGTCCGATTCAGTCATGCCCGCGACCCCATCTTCGGCAACCAGATCATCCCCGACACCGCTATCCTCAGCGTGGTGCCATTTCACCACGGCTTCGGCATGTTCACCACGCTGGGCTACTTGATCTGCGGCTTTCGGGTCGTGCTCATGTACCGCTTCGAGGAGGAGCTATTCTTGCGCAGCTTGCAAGACTATAAGATTCAATCTGCCCTGCTGGTGCCCACACTATTTAGCTTCTTCGCTAAGAGCACTCTCATCGACAAGTACGACCTAAGCAACTTGCACGAGATCGCCAGCGGCGGGGCGCCGCTCAGCAAGGAGGTAGGTGAGGCCGTGGCCAAACGCTTCCACCTACCAGGCATCCGCCAGGGCTACGGCCTGACAGAAACAACCAGCGCCATTCTGATCACCCCCGAAGGGGACGACAAGCCTGGCGCAGTAGGCAAGGTGGTGCCCTTCTTCGAGGCTAAGGTGGTGGACTTGGACACCGGTAAGACACTGGGTGTGAACCAGCGCGGCGAGCTGTGCGTCCGTGGCCCCATGATCATGAGCGGCTACGTTAACAACCCCGAGGCTACAAACGCTCTCATCGACAAGGACGGCTGGCTGCACAGCGGCGACATCGCCTACTGGGACGAGGACGAGCACTTCTTCATCGTGGACCGGCTGAAGAGCCTGATCAAATACAAGGGCTACCAGGTAGCCCCAGCCGAACTGGAGAGCATCCTGCTGCAACACCCCAACATCTTCGACGCCGGGGTCGCCGGCCTGCCCGACGACGATGCCGGCGAGCTGCCCGCCGCAGTCGTCGTGCTGGAACACGGTAAAACCATGACCGAGAAGGAGATCGTGGACTATGTGGCCAGCCAGGTTACAACCGCCAAGAAGCTGCGCGGTGGTGTTGTGTTCGTGGACGAGGTGCCTAAAGGACTGACCGGCAAGTTGGACGCCCGCAAGATCCGCGAGATTCTCATTAAGGCCAAGAAGGGCGGCAAGATCGCCGTGTAA

**nanoLuciferase (nLuc; from pNL1.1)**

ATGGTCTTCACACTCGAAGATTTCGTTGGGGACTGGCGACAGACAGCCGGCTACAACCTGGACCAAGTCCTTGAACAGGGAGGTGTGTCCAGTTTGTTTCAGAATCTCGGGGTGTCCGTAACTCCGATCCAAAGGATTGTCCTGAGCGGTGAAAATGGGCTGAAGATCGACATCCATGTCATCATCCCGTATGAAGGTCTGAGCGGCGACCAAATGGGCCAGATCGAAAAAATTTTTAAGGTGGTGTACCCTGTGGATGATCATCACTTTAAGGTGATCCTGCACTATGGCACACTGGTAATCGACGGGGTTACGCCGAACATGATCGACTATTTCGGACGGCCGTATGAAGGCATCGCCGTGTTCGACGGCAAAAAGATCACTGTAACAGGGACCCTGTGGAACGGCAACAAAATTATCGACGAGCGCCTGATCAACCCCGACGGCTCCCTGCTGTTCCGAGTAACCATCAACGGAGTGACCGGCTGGCGGCTGTGCGAACGCATTCTGGCGTAA

**RT-qPCR primers**

F_pGL4.13 RT-qPCR: GCAGTACCGGATTGCCCAAG

R_pGL4.13 RT-qPCR: GTCGGGGATGATCTGGTTGC

F_nLuc (pNL1.1) RT-qPCR: CAGCGGGCTACAACCTGGAC

R_nLuc (pNL1.1) RT-qPCR: AGCCCATTTTCACCGCTCAG
